# A BTO/PVDF/PDMS Piezoelectric Tangential and Normal Force Sensor Inspired by a Wind Chime

**DOI:** 10.3390/mi14101848

**Published:** 2023-09-27

**Authors:** Chunyan Zhang, Xiaotian Zhang, Qiang Zhang, Shengbo Sang, Jianlong Ji, Runfang Hao, Yan Liu

**Affiliations:** 1 Shanxi Key Laboratory of Micro Nano Sensors & Artificial Intelligence Perception, College of Electronic Information and Optical Engineering, Taiyuan University of Technology, Taiyuan 030024, China; zhangcy0680@163.com (C.Z.); zhangqiang01@tyut.edu.cn (Q.Z.); sunboa-sang@tyut.edu.cn (S.S.); jianlongji@yeah.net (J.J.); haorunfang@tyut.edu.cn (R.H.); 2 School of Software, Taiyuan University of Technology, Taiyuan 030024, China; 3 School of Electronic Information, Hangzhou Dianzi University, Hangzhou 310018, China; zxt406081@163.com; 4 Key Lab of Advanced Transducers and Intelligent Control System of the Ministry of Education, Taiyuan University of Technology, Taiyuan 030024, China; 5 Shanxi Research Institute of 6D Artificial Intelligence Biomedical Science, Taiyuan 030031, China

**Keywords:** three-dimensional force detection, pressure sensor, flexible device, piezoelectric effect

## Abstract

There is a growing demand for flexible pressure sensors in environmental monitoring and human–robot interaction robotics. A flexible and susceptible sensor can discriminate multidirectional pressure, thus effectively detecting signals of small environmental changes and providing solutions for personalized medicine. This paper proposes a multidimensional force detection sensor inspired by a wind chime structure with a three-dimensional force structure to detect and analyze normal and shear forces in real time. The force-sensing structure of the sensor consists of an upper and lower membrane on a polydimethylsiloxane substrate and four surrounding cylinders. A piezoelectric hemisphere is made of BTO/PVDF/PDMS composite material. The sensor columns in the wind chime structure surround the piezoelectric layer in the middle. When pressure is applied externally, the sensor columns are connected to the piezoelectric layer with a light touch. The piezoelectric hemisphere generates a voltage signal. Due to the particular structure of the sensor, it can accurately capture multidimensional forces and identify the direction of the external force by analyzing the position of the sensor and the output voltage amplitude. The development of such sensors shows excellent potential for self-powered wearable sensors, human–computer interaction, electronic skin, and soft robotics applications.

## 1. Introduction

Wearable devices with haptic orientation sensitivity are of interest for their non-destructive, sustainable, and highly accurate orientation detection in various applications, such as human–machine interfaces and environmental health detection sensors [1,2,3,4,5]. Specifically, it is critical to equip sensors with the ability to detect and differentiate between normal and shear forces in real time to enable slip detection and more sophisticated control operations during object interaction.

In recent years, multi-directional force sensors have made impressive progress and have become an essential innovation in engineering and scientific research [6,7,8]. Their high accuracy and sensitivity make them widely used in many fields. Through structural optimization and the continuous development of material technology [9,10,11,12,13], multi-directional force sensors can accurately measure and sense minute forces in all directions, bringing great potential for applications in industrial automation [14], medical diagnosis [15,16], and motion analysis [17,18,19]. Various wearable tactile sensors based on different sensing mechanisms have been widely noticed and reported, which include piezoresistive [20,21], capacitive [6,22], piezoelectric [23,24,25,26,27], and triboelectric [28,29]. Among them, piezoelectric sensors achieve force detection and measurement by utilizing the unique properties of piezoelectric materials to generate charge or voltage signals when subjected to external forces. Their advantages include high sensitivity, fast response, wide measurement range, and durability. Piezoelectric sensors still face some challenges that practical applications need to address. Firstly, structural optimization is necessary to improve the sensors’ accuracy and stability [9,10,12]. Improving the structure of the sensor can reduce the motion and friction of the sensor’s internal components, increasing the sensor’s durability and stability and enabling multi-axis or multi-dimensional measurement capabilities. Secondly, material proportioning is also a key research area. Finding suitable piezoelectric materials, or improving existing materials, can increase the sensitivity and reliability of the sensor while reducing cost and power consumption [11,13].

As an outstanding representative of wearable electronics, piezoelectric material shows unlimited development prospects [30,31,32]. It has become a pioneering material in wearable electronics with its unique advantages of high flexibility, light weight, bio-compatibility, and excellent piezoelectric properties. Various flexible piezoelectric pressure sensors based on polyvinylidene fluoride (PVDF) and its copolymers have been proposed to construct superior pressure sensors [23,25,26]. However, due to their relatively low piezoelectric coefficients, the performance of pressure sensors is limited. Against this background, barium titanate materials (BTO) have attracted much attention and have a promising future due to their higher piezoelectric coefficient, lead-free nature, simple preparation, easy processing, and low cost compared to other piezoelectric ceramic materials. Introducing BTO materials is expected to improve the performance of piezoelectric polymer sensors and provide them with even more excellent performance in the wearable field [24,27]. In addition to the choice of material, the design of the microstructure also plays a significant role in the output of the flexible pressure sensor. Several methods have been proposed to fabricate 3D polymer composites with multidirectional sensing properties, including dome-shaped [6,33,34], pyramidal [12,21], and columnar microstructural patterns [35,36]. Choi et al. [12] designed a pyramidal plug structure, where different types of external mechanical loads produce different deformation mechanisms, enabling it to detect pressure, shear, and torsion. Xu et al. [34] developed an entirely soft capacitive omnidirectional tactile sensor based on MWCNTs coated stripe electrodes and the Ecoflex hemispherical array dielectric structure. The sensing structure, combined with micro-spikes and a hemispherical mound dielectric structure, can achieve high sensitivity and a wide response range of omnidirectional detection. Huang et al. [35] reported a dual-layer contact-resistive structure with a flexible interdental electrode as the active layer, a normal distribution column array constructed by aerosol printing, and a thin-film contact layer fabricated by the sandpaper inversion method. The designed flexible pressure sensor has high sensitivity and fast response time. These innovative designs provide new ways to improve the performance of the sensors and give us a broader outlook on flexible pressure sensing technology.

**Figure 1:**
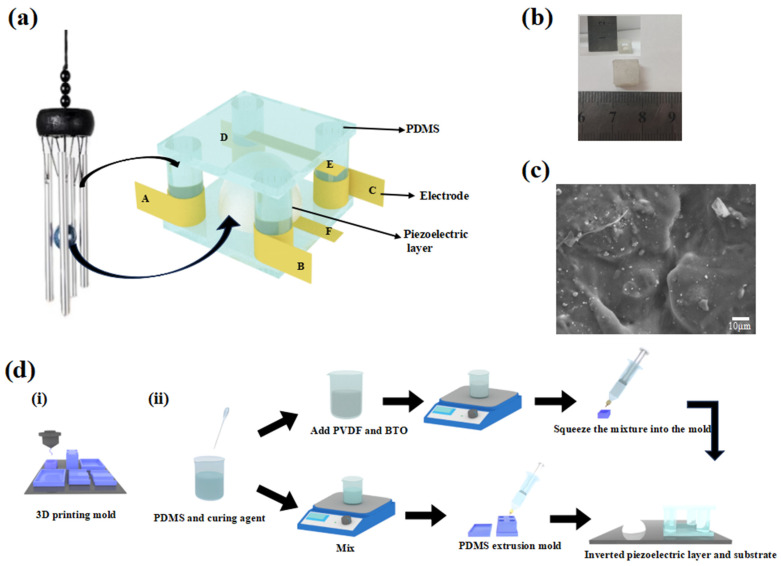
(**a**) Schematic diagram of a 3D sensor with a wind chime structure. In the 3D diagram, A–F represent the electrode labels. (**b**) Physical image of the sensor. (**c**) Scanning electron microscope image of the cross-section of the piezoelectric hemisphere. (**d**) Process flow diagram of the 3D force detection sensor (i) Mold preparation; (ii) Preparation of hemispherical piezoelectric layers with an external substrate.

In this study, we present a piezoelectric 3D force sensor that mimics the structure of a wind chime, as shown in Figure 1a, where piezoelectric layers, similar to the hanging posts on a wind chime, surround the sensor columns. These PDMS columns are precisely arranged so that they can sense forces from all directions. When external forces from different directions act on the sensor columns, they transfer the forces to the piezoelectric layer to produce a piezoelectric effect through a force conduction mechanism, accurately reflecting the direction and magnitude of the external forces. The core building block of the sensor is a hemispherical piezoelectric layer, similar to the center of a wind chime. When subjected to an external force, this piezoelectric material layer can generate an electric charge, converting mechanical energy into electrical energy. Furthermore, we applied periodic external forces in the X, Y, and Z axes and measured the output voltages between AB, CD, AD, BC, and EF, respectively. By analyzing the magnitude of the voltage output, we determined the direction of the force applied to the sensor, fully demonstrating its ability to detect and differentiate between normal and shear forces. This sensor’s unique design and excellent function will bring broader possibilities for future applications.

## 2. Materials and Methods

### 2.1. Materials

PVDF (Arkema, Paris, France) was purchased from Suzhou Yilongsheng Energy Technology Co., Ltd. (Suzhou, China). BaTiO_3_ powder was purchased from Quanzhou Qijin New Material Technology Co., Ltd. (Quanzhou, China). The PDMS elastomer was purchased from Dow Corning as Sylgard 184.

### 2.2. Piezoelectric Elastomer Mold Preparation

Figure 1d(i) shows the preparation of the piezoelectric elastomer mold. We prepared the hemispherical piezoelectric elastomer molds with a radius of 2.5 mm and printed the external substrate molds using a 3D printer. The upper part of the substrate mold consists of a rectangle with side lengths of 13 mm and height of 5 mm. At a distance of (±3.5, ±3.5) mm from the center, there are four cylinders with a radius of 1.5 mm. The lower part of the mold is a rectangle with a side length of 15 mm and a height of 4 mm; in the middle of this rectangle, there is a rectangular groove with a side length of 10 mm and a thickness of 1 mm. Above that, there is a groove with a side length of 13 mm and a thickness of 2 mm, which provides slots for the three upper parts of the mold and serves as a fixing mechanism.

### 2.3. Preparation of Substrate

The preparation process of the 3D force detection sensor is shown in Figure 1d(ii). PDMS was mixed with the curing agent in a ratio of 10:1 using a magnetic stirrer at 300 rpm for 20 min. The mixture was then placed in a vacuum defoamer at 2000 for 2 min to ensure thorough mixing and defoaming of the a-gum and b-gum components. Next, 3g of the PDMS mixture was taken and transferred into an empty 10 mL injection tube. The mixture was then squeezed into the prepared cylindrical substrate mold. The mold with the PDMS mixture was placed in an electric blast drying oven (produced by Shanghai Yiheng Scientific Instrument Co., Ltd., Shanghai, China) and heated at 80 °C for 40 min to allow the PDMS to fully solidify. Carefully, the PDMS cylindrical substrate was removed from the mold. Similarly, a square PDMS film with a thickness of 1 mm and a side length of 10 mm was fabricated. A small amount of PDMS mixture was applied to the joint between the PDMS cylindrical substrate and the PDMS film to act as an adhesive. After sufficient drying, the assembly was solidified under 80 °C, resulting in a flexible cylindrical substrate. Electrodes were then attached to the top and bottom of the substrate, as well as to the bottom of the four cylinders.

### 2.4. Preparation of Three-Dimensional Force Detection Sensors

The piezoelectric layer was prepared as shown in Figure 1d(ii) by weighing 3 g of PDMS mixture and adding 0.24 g of polyvinylidene fluoride (PVDF) powder and 0.24 g of barium titanate powder (BTO). The mixture was then placed on a magnetic mixer and maintained at 300 rpm for 20 min. The prepared solution was transferred into an injection tube (10 mL), and the piston was pushed to remove any air trapped inside. The internal ceramic suspension was then squeezed into a hemispherical mold with a radius of 2.5 mm using the injection tube. The mold was placed in an oven at 80 °C for 30 min to ensure complete solidification and facilitate the demolding process. Subsequently, the piezoelectric layer was placed at the center of the substrate interior, and the physical 3D force detection sensor was prepared, as depicted in Figure 1b.

### 2.5. Characterization and Measurement

The output sensor voltages were measured using a digital multimeter (Keithley 144 2400, Tektronix, Beaverton, OR, USA). The morphology of the samples was analyzed using a scanning electron microscope (JSM-7900F, Tokyo, Japan). The elemental distribution was also qualitatively tested using a complementary energy spectrometer. For the cyclic pressure release experiment, a universal tensile testing machine (produced by Wise Precision Instruments Co., Ltd., model ZQ-990, Dongguan, China) was utilized. The universal tensile machine loading module was used to measure the force from different directions during the movement of the acrylic plate.

## 3. Results and Discussion

**Figure 2:**
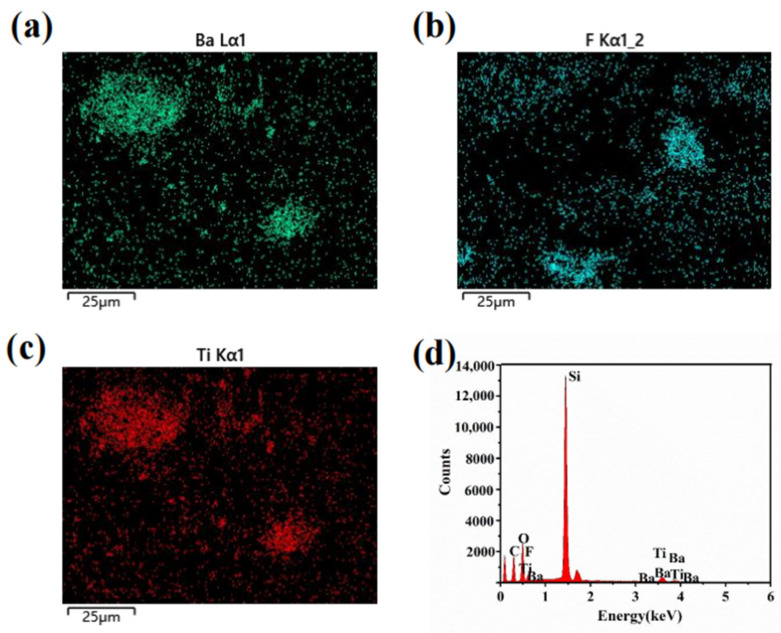
(**a**–**c**) Mapping images of F, Ba, and Ti elements in the BTO/PVDF/PDMS piezoelectric elastomer. (**d**) SEM image of the PVDF/BTO/PDMS composite membrane.

The fabrication process of the flexible 3D force detection sensor is illustrated in Figure 1d. The proposed sensor consists of a piezoelectric layer, a PDMS external substrate, and four cylindrical bottom ends of the substrate with electrodes attached on the top and bottom for output, as depicted in Figure 1a. The highly flexible piezoelectric layer is positioned within the substrate to enhance the long-term durability of the sensor through repeated contact and separation cycles with the electrodes. The physical view of the flexible 3-dimensional force detection sensor is presented in Figure 1b. Additionally, Figure 1c displays a scanning electron microscopy image of the cross-section of the piezoelectric hemisphere, demonstrating the uniform dispersion of PVDF and BTO within the prepared PDMS piezoelectric hemisphere. Energy spectrum analysis conducted using an energy spectrometer reveals that the surface of the hemisphere comprises Ba, F, and Ti elements, as shown in Figure 2a–c. Furthermore, the EDS energy spectrum in Figure 2d demonstrates that the piezoelectric hemisphere consists of PVDF, BTO, and PDMS, representing the three materials present.

### 3.1. Detection of Normal Force

**Figure 3:**
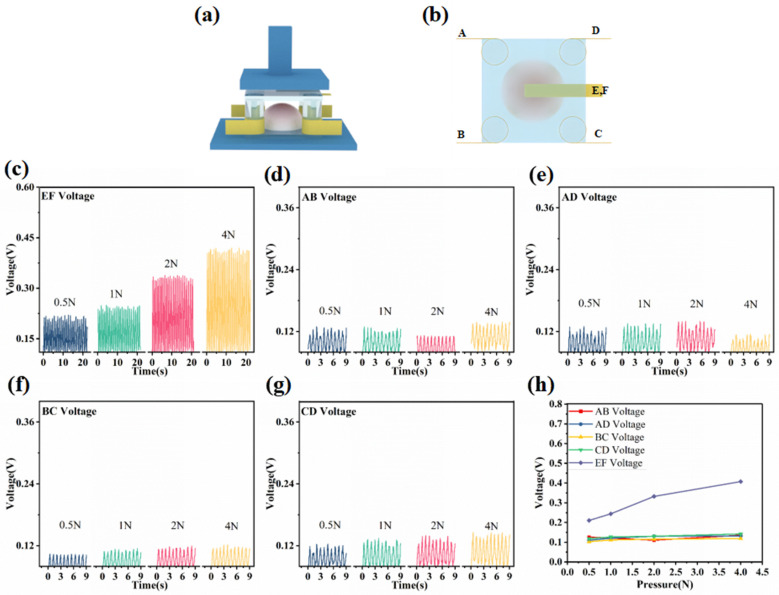
Output performance of the sensor under normal force. (**a**) Schematic diagram of the *z*-axis normal force applied to the sensor. (**b**) Plot of the force position on the sensor. (**c**–**g**) Voltage outputs of AB, AD, BC, CD, EF under a normal force ranging from 0.5 N to 4 N applied to the sensor, respectively. (**h**) Dot plot of the peaks of the waveforms of the output voltages between each pair of electrodes AB, AD, BC, CD, EF, averaged over the average value of the waveforms.

A normal force ranging from 0.5 N to 4 N was applied, and the voltage signal responses of AB, AD, BC, CD, and EF were collected and analyzed. Figure 3a illustrates the simulation of the mechanically applied force on the 3D force sensor placed on the platform. The normal force is applied to the top part of the hemisphere, as depicted in Figure 3b. The sensor is subjected to a normal force ranging from 0.5 N to 4 N. The voltage outputs of AB, AD, BC, CD, and EF are presented in Figure 3c–g. It is evident from the figures that the output voltage of electrode EF exhibits a significant growth trend, while the output voltages of electrodes AB, AD, BC, and CD remain unchanged. This can be attributed to the downward normal force applied to the piezoelectric layer, causing periodic separation between the upper electrode level E and the top of the hemispherical piezoelectric layer, while the bottom of the hemisphere remains in contact with the lower electrode F. The compression of the top of the hemispherical piezoelectric layer under the application of the normal force leads to a substantial increase in the output voltage.

In contrast, the remaining electrodes do not undergo direct squeezing action, resulting in smaller variations in their output voltages compared to EF. To provide a more intuitive comparison, we calculate the average value based on the peak values of the output voltage between the electrodes, as depicted in Figure 3h. This visualization clearly shows that the output voltage of EF is the highest. On the other hand, the other voltage outputs exhibit consistent behavior, allowing us to determine whether the sensor is subjected to the normal force.

### 3.2. Detection of Shear Force

**Figure 4:**
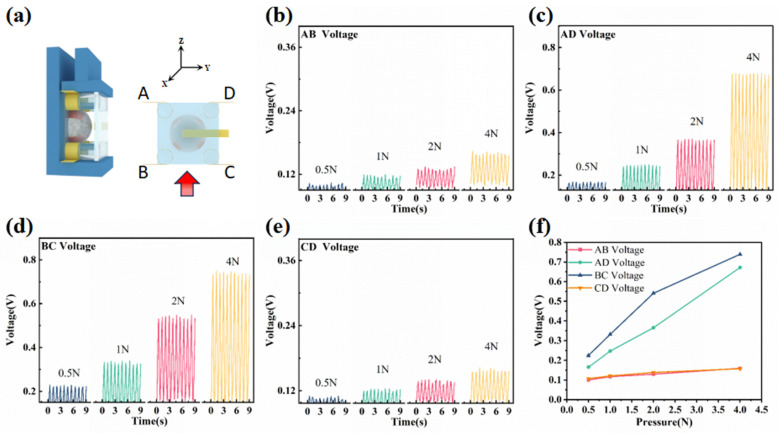
Output performance of the sensor under *x*-axis shear force: (**a**) Schematic diagram of the *x*-axis shear force applied to the sensor and the force position. (**b**–**e**) Voltage outputs of AB, AD, BC, and CD under a shear force ranging from 0.5 N to 4 N applied to the sensor, respectively. (**f**) Dot plot of the peaks of the output voltages of electrodes AB, AD, BC, and CD averaged over time.

The sensor is positioned on the universal tensile machine, as illustrated in Figure 4a, to cyclically apply a force ranging from 0.5 N to 4 N. Figure 4a displays the schematic diagram of the shear force in the *x*-axis. The curves depicting the changes in output voltage under different forces in the *x*-axis are presented in Figure 4b–e, representing the voltage output curves of electrodes AB, AD, BC, and CD, respectively. It can be observed that when the flexible 3D force sensor is subjected to the *x*-axis force, the external substrate of the piezoelectric layer undergoes deformation, causing the electrodes on the cylinders to displace along the *x*-axis direction and compress the piezoelectric hemisphere. As a result, the output voltages of electrodes BC and AD exhibit an increasing trend, whereas the endpoints of electrodes AB and CD are not directly affected by the force, leading to minimal changes in their trends. The peaks of the output voltage curves between the electrodes are averaged under different forces in the *x*-axis, as depicted in Figure 4f. It is evident that the output voltages of electrodes BC and AD are more pronounced compared to electrodes AB and CD when subjected to the *x*-axis force. Moreover, the output voltages of AB and CD demonstrate a consistent trend. Based on these observations, it can be concluded that the sudden increase and more pronounced output trend of electrodes BC and AD with the increase in force indicate their exposure to shear force in the *x*-axis direction.

**Figure 5:**
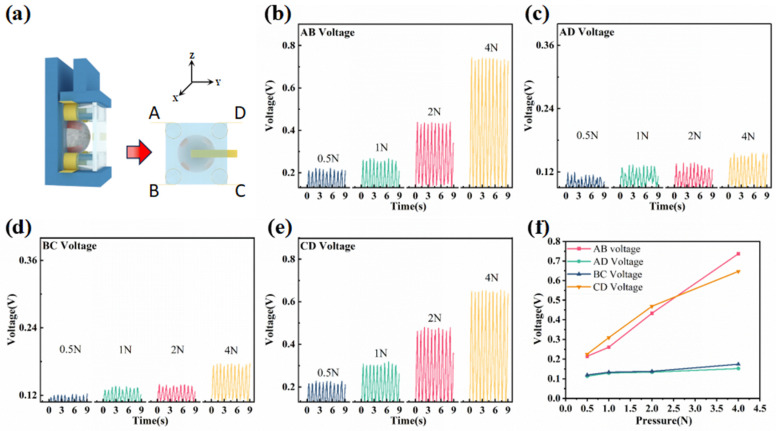
Output performance of the sensor under *y*-axis shear force: (**a**) Schematic diagram of the *y*-axis shear force applied to the sensor and the force position. (**b**–**e**) Voltage outputs of AB, AD, BC, and CD under a shear force ranging from 0.5 N to 4 N applied to the sensor, respectively. (**f**) Dot plot of the peaks of the output voltages of electrodes AB, AD, BC, and CD averaged over the measurements.

The force schematic of the sensor in the *y*-axis is depicted in Figure 5a, where the 3D force sensor is positioned on the platform, and the force direction on the piezoelectric hemisphere under the *y*-axis force is towards electrode AB. Figure 5b–e illustrate the output voltage profiles of electrodes AB, AD, BC, and CD under a dynamic *y*-axis force ranging from 0.5 N to 4 N. When the *y*-axis force is applied, the deformation of the substrate causes displacement of the cylinder along the *y*-axis direction, resulting in the compression of the piezoelectric hemisphere by electrodes AB and CD on the cylinder. Consequently, the output voltages of electrodes AB and CD exhibit a more pronounced growth trend, while electrodes BC and AD, not directly influenced by the force, show less noticeable changes in their output voltages. The peaks of the output voltage curves presented in Figure 5b–e are averaged and plotted in Figure 5f, clearly demonstrating the apparent growth trend in the output voltages of electrodes AB and CD. Conversely, the output voltages of electrodes BC and AD remain relatively unchanged compared to electrodes AB and CD. It can be observed that, when the output voltage between electrodes BC and AD of the flexible three-dimensional force sensor is higher than that of the other electrodes and exhibits an increasing trend with the magnitude of the force, it can be inferred that the sensor is subjected to the *y*-axis force at that time.

By conducting a comprehensive analysis of the relevant literature, as illustrated in Table 1, it becomes evident that sensors employing a hemispherical structure as the sensing layer offer significant advantages. The spherical design of these sensors enables them to achieve uniform measurement of multidirectional forces. In contrast, sensors with cylindrical and pyramidal microstructures may require further optimization to enhance their multidirectional measurement capabilities due to their unique structural shapes. Moreover, the hemispherical structure facilitates an even distribution of forces in all directions, thereby reducing the likelihood of stress concentration and enhancing both the accuracy and reliability of measurements. Furthermore, a comparison of the detection ranges presented in Table 1 demonstrates that sensors featuring a hemispherical structure as the sensing layer exhibit superior structural strength, enabling them to withstand higher forces and stress levels. In conclusion, sensors with a hemispherical structure as the sensing layer clearly demonstrate their superiority in terms of multidirectional force measurement, uniform stress distribution, and structural strength. Additionally, when examining the functionality achieved, this paper’s sensor stands out among the related literature, as it successfully detects multidirectional forces and offers a wide detection range (approximately 64–1770 kPa).

## 4. Conclusions

In this paper, we propose the design of a highly flexible wind-chime-structured multidirectional force piezoelectric sensor. The sensor incorporates electrodes and a sensing layer designed in a contact separation mode, which enhances its long-term durability. This design enables its application in physiological information detection and action type recognition. The sensor comprises an external substrate supported by four PDMS cylinders and a hemispherical piezoelectric elastomer made of a BTO/PVDF/PDMS composite material. This configuration offers excellent performance with a wide detection range (approximately 64–1770 kPa) and the ability to detect and differentiate between normal and shear forces. When pressure is applied in the *z*-axis direction, the voltage across the EF electrode of the sensor increases rapidly. Similarly, the voltages across the AB and CD terminals increase rapidly when pressure is applied in the *y*-axis direction, and the voltages across the AD and BC terminals increase rapidly when pressure is applied in the *x*-axis direction. By analyzing these different output voltages, we can accurately determine the direction of the force applied to the sensor. Additionally, by utilizing the output voltages from different electrodes, we can accurately assess the sliding condition of objects manipulated by the sensor. Looking ahead, our future research will focus on the miniaturization and array design of sensors to achieve more sophisticated intelligent tactile sensing capabilities. These advancements will enable robots to perceive dimensional force information more effectively, leading to their improved performance and responsiveness in various situations.

## Figures and Tables

**Table 1:** Comparison of design and functionality with other pressure sensors.

Mechanism	Design	Multidimensional Force	Pressure Range	Ref.
capacitive	pyramida	yes	0–50 kPa	[12]
	dome-shaped	yes	2.55–160 kPa	[34]
triboelectricity	microcolumn	no	0.04–1200 kPa	[36]
piezoresistive	pyramida	yes	0~225 Pa	[21]
	columnar	no	0–128 kPa	[35]
	dome-shaped	no	0–12 kPa	[33]
piezoelectric	dome-shaped	no	0–255 kPa	[23]
	dome-shaped	yes	≈64–1770 kPa	This work

## Data Availability

The experimental data are available from the authors.
